# Secondary Metabolites from a New Antibiotic-Producing Endophytic Streptomyces Isolate Inhibited Pathogenic and Multidrug-Resistant *Mycobacterium tuberculosis* Strains

**DOI:** 10.3390/tropicalmed10050117

**Published:** 2025-04-23

**Authors:** Govinda Raju Vadankula, Arshad Rizvi, Haider Ali, Rakhi Khunjamayum, V. V. Ramprasad Eedara, Vijay Nema, Debananda Singh Ningthoujam, Katragadda Suresh Babu, Prakasham Reddy Shetty, Shekhar C. Mande, Sharmistha Banerjee

**Affiliations:** 1 Laboratory of Molecular Pathogenesis, Department of Biochemistry, School of Life Sciences, University of Hyderabad (UoH), Hyderabad 500046, India; govindraj.vadankula@gmail.com (G.R.V.); arshad.rizvi@emory.edu (A.R.); ali.haider@doctoral.uj.edu.pl (H.A.); 2 Microbial Biotechnology Research Laboratory (MBRL), Department of Biochemistry, Manipur University, Canchipur 795003, India; rakhikhunjamayum@gmail.com (R.K.); debananda.ningthoujam@gmail.com (D.S.N.); 3 MNR Medical College and Hospital, MNR Nagar, Fasalwadi, Narsapur Road, Sanga Reddy, Hyderabad 502294, India; ramprasadevv@gmail.com; 4 Molecular Biology Division, National Institute of Translational Virology and AIDS Research (Formerly National AIDS Research Institute), Pune 411026, India; dr.vijaynema@gmail.com; 5 Centre for Natural Products and Traditional Knowledge, CSIR-Indian Institute of Chemical Technology, Hyderabad 500007, India; suresh@iict.res.in; 6 Medicinal Chemistry and Biotechnology Lab- Organic Synthesis and Process Chemistry Division, CSIR-Indian Institute of Chemical Technology, Hyderabad 500007, India; prakasham@iict.res.in; 7 National Centre for Cell Science, Pune 411007, India; shekhar.mande@gmail.com; 8 Bioinformatics Centre, Savitribai Phule Pune University, Pune 411007, India

**Keywords:** actinomycetes, antibiotics, tuberculosis, anti-*M.tb* activity, MDR-TB, LC-MS

## Abstract

The long regimen of drug therapy, the emergence of drug-resistance (DR), and infections with non-tuberculous mycobacteria (NTMs) are alarming challenges in controlling tuberculosis (TB), a disease caused by *Mycobacterium tuberculosis* (*M.tb*), necessitating the pursuit of new, broad-spectrum anti-mycobacterials. With more than two-thirds of the clinically useful antibiotics originating from the bacterial phylum Actinomycetota, and their enormous diversity in India, we explored atypical environments for new bacterial strains with potential anti-*M.tb* activity. In this study, we the examined the secondary metabolites of soil and endophytic bacterial isolates from the wetland niches of Manipur, India, and determined their anti-mycobacterial properties using viability assays. The ethyl acetate culture filtrate extracts of one of the isolates, named *Streptomyces* sp. SbAr007, showed broad-spectrum anti-mycobacterial activity against laboratory *M.tb* strains H37Ra and H37Rv, a clinical drug-resistant *M.tb* and non-tuberculous mycobacteria (NTM). The isolate was characterized for its phenotype and genetic identity, which indicated its closeness to *Streptomyces samsunensis*, *Streptomyces malaysiensis*, and *Streptomyces solisilvae*. Further, macrophage infection assays showed that the extracts could effectively control the intracellular mycobacterial growth but had negligible cytotoxicity to PBMCs from healthy donors. LC-MS identified an unusual combination of antibiotics in these culture filtrate extracts, which can be further explored for specific active molecules or as a formulation against DR-TB.

## 1. Introduction

*Mycobacterium tuberculosis* (*M.tb*) is the agent responsible for the disease tuberculosis (TB) [1], which, despite being curable, has caused 1.3 million deaths across the world in 2022 [2] and infected 10.8 million people globally in 2023 [3], suggesting its co-evolution with the hosts undergoing the present drug treatments [4]. TB treatment is categorized into first- and second-line treatments. The first-line treatment, which is generally followed for newly diagnosed drug-sensitive TB, includes rifampin, isoniazid, pyrazinamide, and ethambutol (RIPE), which has an intensive phase of 2 months and, subsequently, a prolonged treatment phase of 4–7 months [5]. This long and drug-burdened treatment often leads to non-compliance, which is evident from the dramatic increase in drug-resistant (DR) TB. The WHO reported that about 0.4 million people have developed DR-TB (including multidrug-resistant (MDR)-TB and rifampicin-resistant (RR)-TB), and India alone accounted for 27% of the DR-TB cases over the past three years (from 2020 to 2023) [3]; this report explains the emergence of DR-TB. DR-TB requires treatment with the second-line drugs, which comprise fluoroquinolones, oxazolidinone, such as linezolid, injectable broad-spectrum antibiotics [6], with pretomanid and bedaquiline being recently added [7]. However, the alarming part is the emergence of resistance not only confined to first-line drugs (MDR-TB) but also to second-line drugs (extensively drug-resistant TB or XDR-TB), which leaves very few options for treatments. Bacille Calmette-Guérin (BCG) is the only available TB vaccine that can protect children effectively, but it cannot protect adults, especially patients with comorbidities. To address this, several TB vaccine studies are being employed which resulted in development of 6 TB vaccines that are currently in phase III clinical trials; however, they are not yet available [3]. In addition, infections by non-tuberculous mycobacteria are on the rise, most commonly with pulmonary infections, especially in immunocompromised individuals [8,9]. This rationalizes the search for new chemical entities, either through natural or synthetic sources with broad-spectrum activity. Interestingly, the most successful anti-TB drug, rifampin, is a semi-synthetic version of a metabolite, rifamycin, isolated from a soil Gram-positive bacteria *Amycolatopsis rifamycinica* [10,11], referred to as *Streptomyces mediterranei* in some studies [11,12,13]. Also, drugs like cycloserine, which is used for MDR-TB, were a natural derivative of *Streptomyces orchidaceus* in the 1950s. With these illustrations, a continuous search for natural metabolites with anti-*M.tb* activity becomes highly promising in the fight against emerging drug resistance in TB.

Among the various natural antibiotic producers, microorganisms such as bacteria and fungi still are a good source of antibiotics [14,15]. As we know, the majority of the known antibiotics produced thus far are from actinomycetes [16] and actinomycetes have been helpful, particularly for the unlimited source of novel secondary metabolites [17,18]. Yet another important point for natural antibiotic discovery is to explore atypical and extreme ecological niches. With a limited understanding of the bacterial diversity in these niches, the exploration of bioactive molecules has also been inadequate. Northeast India is known for its distinct ecological diversity; it is important that these microbial communities must be explored. This study is a collaborative effort wherein microbial isolates from atypical environments, including endophytes from Manipur, India, were investigated for their anti-mycobacterial properties. Here, we report a new isolate of actinomycetes with anti-mycobacterial activity. This isolate, named SbAr007, was found to be close to streptomyces species *S. malaysiensis*, *S. solisilvae*, and *S. samsunensis*, as per 16S rRNA gene sequencing (GenBank ID: KY681811.1). The solvent (ethyl acetate)-extracted growth-culture filtrates showed anti-mycobacterial activity against various pathogenic and non-pathogenic mycobacteria. It showed an MIC_50_ (the growth inhibitory concentration at which 50% of the bacteria is viable or dead) of 5.15 μg/mL for virulent *M.tb* strain H37Rv and 2.49 μg/mL for avirulent strain H37Ra using a 3-(4,5-dimethylthiazol-2-yl)-2,5-diphenyltetrazolium bromide (MTT) assay. Interestingly, the extracts were also active against a clinical MDR-*M.tb* isolate and NTMs and also found to be effective in clearing intracellular mycobacteria in macrophages in vitro. The cytotoxicity on host cells was checked using peripheral blood mononuclear cells (PBMCs) isolated from ten healthy donors. LC-MS was employed to identify the metabolites in the active fractions, which showed a combination of a battery of known antibiotics and other metabolites. The assays showed negligible cytotoxicity, indicating their safety if explored for further drug development. With this, we report a new antibiotic-producing streptomyces isolate, the culture filtrate of which can be further explored for the isolation of new active molecules or can be developed as a formulation against drug-resistance TB.

## 2. Materials and Methods

### 2.1. Bacterial Strains

Actinomycete strains were isolated from different habitats of Manipur, India, such as *Acorus calamus* rhizomes (AcRz3), *Tectona grandis* roots (TgR6 renamed as SbAr007), *Solanum xanthocarpum* (Leaves: SxL4, SxL7, SxL10, SxL11; Stem: SxS3; Roots: SxR1), and *Artemisia nilagirica* roots (ANR10), and cultured as described previously [19]. Briefly, starch casein nitrate agar media was prepared by mixing 1 mg of starch, 0.03 gm of casein, 0.2 gm each of NaCl, KNO_3_, and K_2_HPO_4_, 0.005 gm of MgCl_2_, 0.002 gm of CaCO_3_, and 0.001 gm of FeSO_4_, and the final volume was adjusted to 100 mL with MilliQ water. After the pH was set to 7.25, agar (1.8 gm) was added for preparing SCN–agar media, whereas no agar was added for preparing SCN–broth media, and it was sterilized for 20 min at 120 °C. A 100 µL of actinomycetes culture was inoculated in broth media and incubated in a shaker incubator at 30 °C for 7 to 8 days and processed for culture filtrate extracts isolation.

A non-pathogenic mycobacterial strain, *Mycolicibacterium smegmatis* (basonym *Mycobacterium smegmatis* mc^2^155) (obtained from CDFD, Hyderabad), and non-tuberculosis mycobacterial strains, *M. phlei* (MTCC 1724) and *M. fortuitum* (MTCC 1902), were cultured in Middlebrook 7H9 media broth (Himedia, India) supplemented with 2% glycerol (*v*/*v*) and Tween 80 (0.05% *v*/*v*) and incubated in a 37 °C shaker incubator. Mycobacterial studies involving pathogenic strains were conducted in a biosafety level III lab (UoH-NIAB BSL3 facility), University of Hyderabad, Hyderabad, India, as per the approved IBSC guidelines. Investigations with clinical strains were carried out at the biosafety level III lab, NITVAR, Pune, as per the approved IBSC guidelines. BSL level III mycobacterial strains, such as *M.tb* H37Rv (obtained from JALMA, Agra), avirulent *M.tb* strain H37Ra (obtained from JALMA, Agra), and clinical *M.tb* strain (referred as CH56) (at NITVAR, Pune), were used in this study. Virulent and avirulent mycobacterial strains were cultured as previously described [20]. Concisely, these *M.tb* strains were cultured in Middlebrook 7H9 media broth (Himedia, India) supplemented with 10% OADC (Himedia, India), 0.5% glycerol (*v*/*v*), and Tween 80 (0.05% *v*/*v*), and incubated at a 37 °C shaker incubator. All the cultures were confirmed contamination-free using the Ziehl–Neelsen staining kit (Himedia, India).

### 2.2. Ethyl Acetate Extraction of Culture Filtrate Extracts from Actinomycetes Strains

Fully grown actinomycetes cultures were harvested via centrifugation and collected the filtered supernatant in a fresh flask. A total of 100 mL of ethyl acetate was added to one liter of culture supernatant and mixed vigorously by shaking and resting for 2 and 1 min, respectively. This step was repeated ten times, and the ethyl acetate solvent was collected using a separating funnel. A fresh 100 mL of ethyl acetate was added to the same culture supernatant, and the whole extraction procedure was repeated twice and pooled all the ethyl acetate fractions. The pooled ethyl acetate fractions were concentrated using rota-evaporation. The concentrated extracts were stored at −20 °C until further processes.

### 2.3. Genomic DNA Isolation and PCR Amplification of 16S rRNA Gene

Genomic DNA from the *Streptomyces* sp. SbAr007 strain was isolated using the GeneJET Genomic DNA purification Kit (Thermo Fisher Scientific, Waltham, MA, USA) as per the manufacturer’s instructions. Briefly, a 5 mL culture pellet was resuspended in Gram-positive bacteria lysis buffer (20 mM Tris-HCl, pH 8.0, 2 mM EDTA, 1.2% Triton X-100, and lysozyme (20 mg/mL)) and incubated at 37 °C for 30 min. We added lysis solution containing RNase A and proteinase K and activated proteinase K via incubation at 56 °C for 30 min. The genomic DNA was eluted in the elution buffer after subsequent washes of cell lysate with 50% ethanol, wash buffer I, and wash buffer II. The isolated genomic DNA was used as a template to amplify its 16S rRNA gene. Universal ribosomal DNA primers F-27 (FP: AGAGTTTGATCMTGGCTCAG) and R-1494 (RP: CTACGGYTACCTTGTTACGAC) [21] were synthesized and used in the PCR. We amplified the 1.4Kb of 16S rRNA gene using a Taq polymerase, with the following PCR conditions: initial denaturation at 94 °C for 2 min, followed by cyclic denaturation at 94 °C for 30 s, allowed to anneal at 55 °C for 30 s, followed by cyclic extension at 68 °C for 90 s for 35 cycles. A final extension and final hold were setup at 68 °C for 10 min and 4 °C, respectively, to amplify the 16S rRNA gene. The amplicon was run on a 1% agarose gel, and the 16S rRNA gene was sequenced. The sequence was deposited to NCBI with a GenBank ID: KY681811.1.

### 2.4. Mycobacterial Cell Viability Assay

Mycobacterial viability assays were performed using MTT assays in 96-well microtiter plates as described [20]. In summary, primary mycobacterial cultures (*M. tuberculosis* H37Ra, *M. tuberculosis* H37Rv, and *M. smegmatis*) were grown in Middlebrook 7H9 broth media (Himedia, India) until an O.D_600_ 0.8 to 1.2 was reached. Then, 5 × 10^5^ mycobacterial cells per well were seeded with the culture filtrate extracts, using DMSO as a vehicle control, and incubated at 37 °C for seven days for all the strains, except *M. smegmatis* and NTMs, which were incubated for 24 h. To assess the viability of cells, after incubation for the mentioned time, 10 μL of 5 mg/mL of MTT (Himedia, India) was added per well, as previously described [20]. Further the change in the color was recorded using a microplate spectrophotometer (Thermo Scientific Multiskan GO, Thermo Fisher Scientific, Waltham, MA, USA) at 570 nm. Corresponding dose–response plots were generated using GraphPad Prism (version 5), and their MIC_50_ (minimum inhibitory concentrations at which 50% of the bacteria is viable or dead) was determined. In preliminary screening, to confirm the anti-mycobacterial properties, a non-pathogenic and fast-growing strain, *M. smegmatis*, was used as a surrogate.

### 2.5. Microplate Nitrate Reductase Assay (MNRA)

MNRA was performed for the clinical MDR-*M. tuberculosis* strain (CH56), as reported earlier [20]. Briefly, KNO_3_ was additionally supplemented along with 7H9 media to mycobacteria for 14 to 21 days with or without the extract. A total of 50 µL of Griess reagent was added per well, and the absorbance at 540 nm was calorimetrically measured. A GraphPad Prism (Version 5) was employed to plot the dose–response curves.

### 2.6. Cytotoxicity Assays in Human Peripheral Blood Mononuclear Cells (hPBMCs)

According to the Institutional Ethics Committee (IEC) guidelines, prior written consent forms from ten healthy volunteers were obtained before collecting the blood (10 mL per volunteer). Isolation of hPBMCs using Histopaque (Merck) and cytotoxicity assays were performed, as previously described [20,22]. Briefly, isolated and quantified hPBMCs were seeded at a density of 0.2 × 10^6^ cells/well in a 96-well plate and treated with a concentration range of 2X to 128X MIC_50_ and incubated till 72 h at 37 °C, supplemented with 5% CO_2_. We monitored the developed color (purple) after adding Alamar blue dye and recorded at 540 nm using a multimode reader (SpectraMax, Molecular Devices, San Jose, CA, USA) to obtain the viability plots using GraphPad Prism (Version 5).

### 2.7. Infection Assays

Infection assays were performed as previously described [22]. Concisely, in a 96-well tissue culture plate, approximately 0.25 × 10^5^ monocytic leukemia cell lines (THP-1) were seeded per well and treated with 100 ng/mL of PMA (phorbol 12-myristate 13-acetate) overnight. They were washed thrice with sterile 1X PBS, replenished with fresh RPMI media, and allowed to rest for 12 h to differentiate into macrophages. They were then infected separately with *M. smegmatis*, *M. phlei*, and *M. fortuitum* at a multiplicity of infection (MoI) 20 for four hours without any antibiotics. Next, cells were washed with sterile 1X PBS to remove the extracellular bacteria and supplemented with fresh RPMI media containing antibiotics. The cells were lysed at zero- and six-hour post-infection (HPI) with sterile MilliQ water, and colony-forming units (CFU) were enumerated by plating on Middlebrook 7H10 (Himedia, India) agar media after 3 days of incubation at 37 °C. Enumerated bacteria were plotted using GraphPad Prism (Version 5).

### 2.8. LC-MS Analysis

Samples were prepared by dissolving 2 mg of culture filtrate extract in 100 μL of a mixture of solvents (Acetonitrile:Methanol:Water), mixed in a 50:30:20 ratio. A total of 10 μL of this dissolved sample was injected into a Shimadzu Prominence-I HPLC (Shimadzu Corporation, Kyoto, Japan), interfaced with a Shimadzu triple quadrupole LCMS-8045 mass spectrometer (Shimadzu Corp., Kyoto, Japan). The chromatographic conditions were set as follows; mobile phase A (Acetic Acid (0.04%) in Water) and B (Acetonitrile (100%); methanol is used as a diluent; Shimpack Gist C18 75*4.0 mm, 3 μm (column ID: CLC0181); the flow rate and run time was set to 0.8 mL/minute and 45 m, respectively; the column oven temperature was set to 30 °C; and the sample cooler temperature was set to 15 °C. The MS conditions were used as follows: acquisition time, 45 min; interface, ESI; scan mode, positive and negative; nebulizing gas flow, 3 L/minute; heating gas, 10 L/minute; interface temperature, 300 °C; drying gas flow, 10 L/minute; and DL temperature, 250 °C. The electrospray ionization (ESI)–MS analysis was performed in both positive- and negative-ion modes. Full-scan mass spectra were acquired over a mass range of *m*/*z* 50–2000. The output raw files were then used for the preprocessing module.

#### Preprocessing of Raw LC-MS Data

Mass feature detection and identification were performed using MZmine. The raw LC-MS files were imported and cropped to a retention time range of 0 to 45 min. The peak detection algorithm is used to detect peaks with masses for each sample. A chromatogram builder algorithm, along with deconvolution and deisotoping of peaks, was used to build chromatograms for all samples. HMDB/KEGG databases were used for metabolite identification with mass tolerance 0.001–0.1 Da (Daltons). Then, we exported the resulting data in CSV format for further downstream analysis.

## 3. Results and Discussion

### 3.1. An Endophytic Strain with Anti-Mycobacterial Properties Isolated from Tectona Grandis

**Figure 1:**
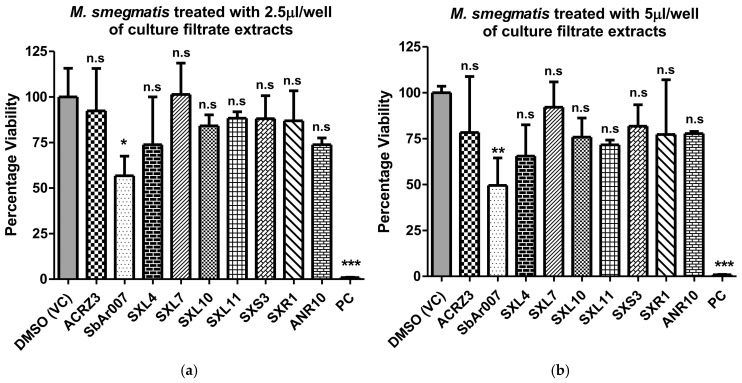
Preliminary MTT assays on *M. smegmatis* with culture filtrate extracts from nine different isolates. *M. smegmatis* was treated with culture filtrate extracts for 24 h, and viability assays were performed. The bar plots represent the percentage viability of *M. smegmatis* treated with (**a**) 2.5 µL and (**b**) 5 µL per well of culture filtrate extracts isolated from the isolates. PC: positive control (hygromycin, 50 µg/mL); VC: vehicle control. One-way ANOVA (Dunnet’s multiple comparison test) was performed, and the error bars represent the standard deviation. “***” denotes *p* ≤ 0.001, “**” denotes a *p* ≤ 0.01, “*” denotes *p* ≤ 0.05. n.s: statistically non-significant.

We earlier isolated nine different isolates from four different medicinal plants from the wetland niches in Manipur, India (AcRz3, SbAr007, SxL4, SxL7, SxL10, SxL11, SxS3, SxR1, and ANR10). We cultured them on starch casein nitrogen (SCN) media and extracted the secondary metabolites from their culture supernatants, as described in the methods section. We employed MTT assays for the preliminary screening of the culture filtrate extracts from these isolates against *Mycolicibacterium smegmatis* (*basonym Mycobacterium smegmatis*) as a surrogate for pathogenic mycobacteria. Of the nine isolates screened, ethyl acetate extracts of culture filtrate from *Streptomyces* sp. SbAr007 grown in SCN media showed significant anti-*M. smegmatis* activity (Figure 1). Compared to the culture filtrates from the rest of the isolates and the DMSO-treated control, *Streptomyces* sp. SbAr007 culture filtrate extracts reduced *M. smegmatis* viability by 49.49% and 56.70% for 5 µL and 2.5 µL, respectively (Figure 1). *Streptomyces* sp. SbAr007 was further streaked on the SCN–agar plate, and a single colony was grown in SCN–broth media and frozen for further assays. We further characterized and investigated the anti-*M.tb* activity of *Streptomyces* sp. SbAr007 culture filtrate extracts on pathogenic mycobacteria.

### 3.2. Culture Filtrate Extracts from Streptomyces sp. SbAr007 Showed Potent Anti-M.tb Activity

**Figure 2:**
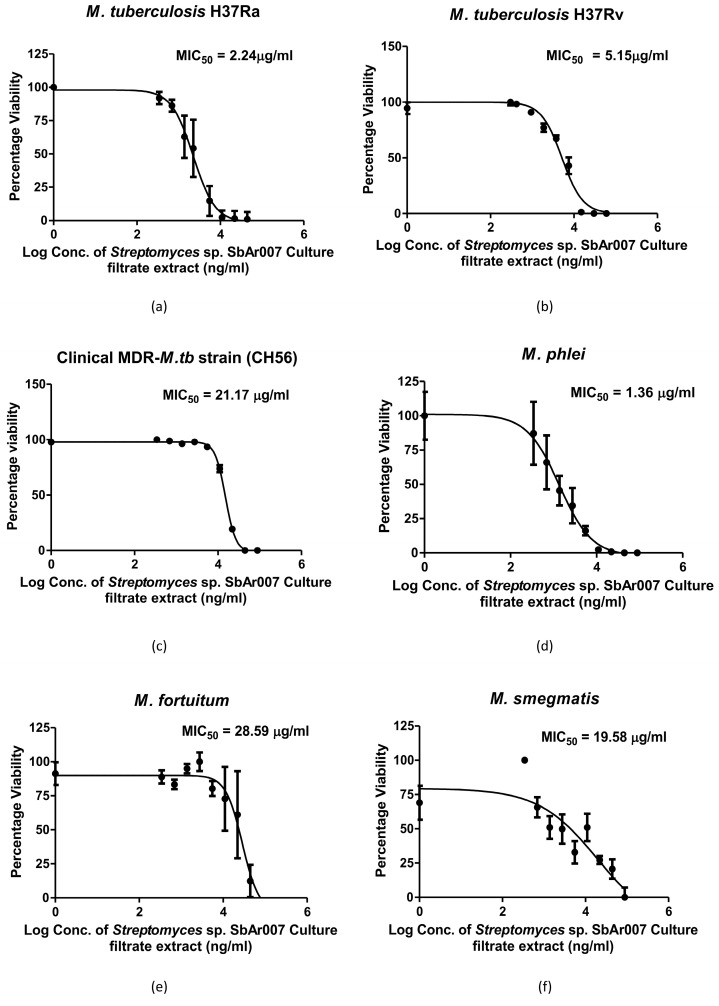
Microdilution assays for determining MIC_50_. Dose–response curves were plotted upon treatment with *Streptomyces* sp. SbAr007 culture filtrate extract against (**a**) *M. tuberculosis* H37Ra, (**b**) *M. tuberculosis* H37Rv, (**c**) a representative clinical MDR-*M. tuberculosis* strain (CH56) (resistant to established anti-TB drugs, i.e., isoniazid and rifampicin), (**d**) *M. phlei*, (**e**) *M. fortuitum*, and (**f**) *M. smegmatis*. All the experiments were performed at least thrice, and the error bars represent the standard deviation.

The culture filtrate of *Streptomyces* sp. SbAr007 grown in SCN–broth media was extracted using the solvent ethyl acetate, as described in methods section, and dissolved in 100% DMSO (referred to as CF extract). Microdilution assays were performed (Supplementary Materials, Figure S1) to determine the minimum inhibitory concentration (MIC) for *M.tb* strains H37Ra, H37Rv, and a representative clinical MDR-*M.tb* strain CH56 resistant to isoniazid (INH) and rifampicin (RIF) (Table S1). Dose–response curves were plotted to determine the MIC_50_, which was found to be 2.24 and 5.15 µg/mL, respectively, for H37Ra and H37Rv (Figure 2a,b). We compared these values with the previously reported studies on anti-H37Rv activities of the plant-derived natural products, which ranged from 0.8 µg/mL to 1600 µg/mL [23]. Our MICs were observed in a similar range or lesser than a few reports on secondary metabolites with anti-*M.tb* activity derived from plants [24,25,26,27,28,29], fungi, and bacteria [30,31,32,33,34]. These observations suggested that the *Streptomyces* sp. SbAr007 culture filtrate extracts had encouraging potency, with low MIC_50_ against laboratory *M.tb* strains. Encouragingly, the MIC_50_ for CH56 was found to be 21.17 µg/mL (Figure 2c).

To investigate if the CF extract can function against non-tuberculous mycobacteria (NTM), microdilution assays were performed for *Mycobacterium phlei* (*M*. *phlei*) and *Mycobacterium fortuitum* (*M*. *fortuitum*). We also included *Mycolicibacterium smegmatis* (*M*. *smegmatis*) as a non-pathogenic saprophytic mycobacterium in this study to assess the spectrum of anti-mycobacterial activity of the CF extract. MIC_50_ values of 1.36 µg/mL, 28.59 µg/mL, and 19.58 µg/mL for *M. phlei*, *M. fortuitum*, and *M. smegmatis*, respectively, were determined (Figure 2d–f). This indicated that the CF extract can work against both *M.tb* and NTMs, suggesting a broad-spectrum anti-mycobacterial activity. Further, with the effective inhibition of the clinical MDR *M.tb* strain, one may envisage that CF extract is also effective against clinical *M.tb* strains.

### 3.3. Culture Filtrate Extract from Streptomyces sp. SbAr007 Showed Negligible Cytotoxicity to Human Peripheral Blood Mononuclear Cells (hPBMCs)

**Figure 3:**
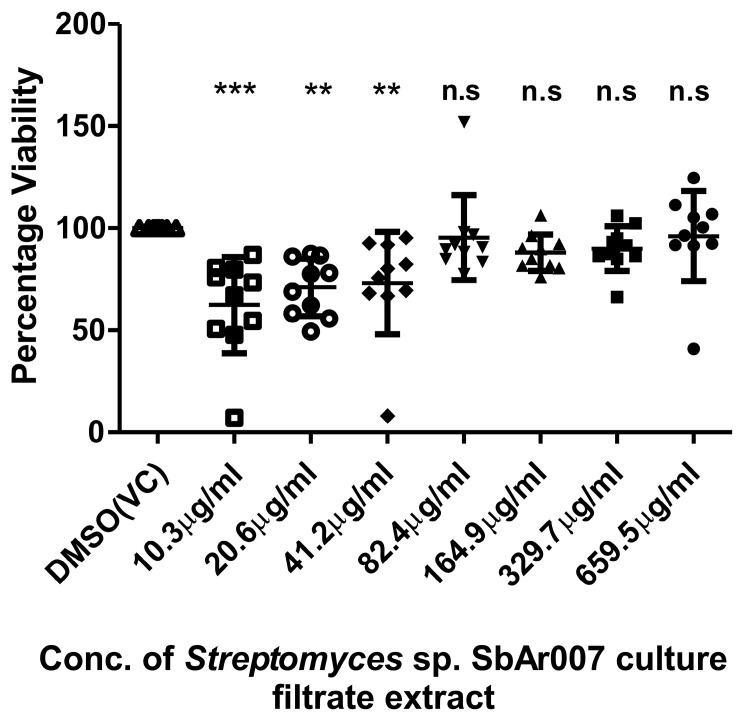
Cytotoxicity of *Streptomyces* sp. SbAr007 culture filtrate extract on isolated hPBMCs in a dose-dependent treatment. VC: vehicle control. One-way ANOVA (Dunnett’s multiple comparison test) was performed, and error bars represent the standard deviation; “***” denotes a *p*-value of < 0.001, “**” denotes *p*-value of <0.01, “n.s” denotes *p*-value > 0.05, and *n* = 10.

With the promising anti-*M.tb* activity of the CF extract, both on clinical and laboratory *M.tb* strains, we further evaluated the cytotoxicity of the CF extract on the isolated hPBMCs from ten healthy volunteers at about 100–120-fold-greater concentrations than the MIC_50_ of H37Rv (5.15 μg/mL). We noted that CF extract had no or negligible cytotoxicity compared to the vehicle control (DMSO) (Figure 3).

### 3.4. Culture Filtrate Extract of Streptomyces sp. SbAr007 Decreased Intracellular Mycobacterial Load

**Figure 4:**
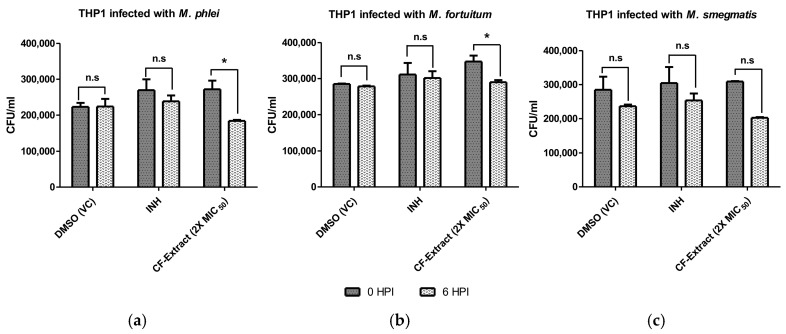
Intracellular clearance of NTMs—(**a**) *M. phlei*, (**b**) *M. fortuitum*, (**c**) *M. smegmatis*—in THP-1 macrophages determined via CFU enumeration. The concentration (1.72, 56, and 40 µg/mL) of culture filtrate (CF) extracts were twice the determined MIC_50_ in *M. phlei*, *M. fortuitum*, and *M. smegmatis*, respectively, and isoniazid (INH) was used as drug control. HPI, hours post infection; VC: vehicle control. Two-way ANOVA (Dunnett’s multiple comparison test) was performed, and the error bars represent the standard deviation; “*” denotes a *p*-value of <0.05, and “n.s” denotes a non-significant *p*-value of > 0.05.

To verify if the anti-mycobacterial impact of the CF extract is also applicable to intracellular mycobacteria, we performed infection assays in differentiated THP-1 (macrophage) cell lines with *M. smegmatis*, *M. phlei*, and *M. fortuitum* and treated the same with two-fold greater concentrations than their respective MIC_50_. THP-1 cells were infected with an MoI of 1:20 for 4 h, followed by adding CF extract until 6 h post-infection (HPI). The infected macrophages were subjected to lysis, lysates from 0-HPI and 6-HPI were plated on 7H10 agar plates, and colony-forming units (CFU) were counted. DMSO was used as a vehicle control, and 0.1 μg/mL of INH was used as a drug control. INH was used as a control since it is known that NTMs are not effectively cleared by INH [35]. The CFU counts between 0-HPI and 6-HPI were compared to assess the clearance of intracellular mycobacteria. The results clearly show that 2X CF extract lowered intracellular mycobacterial load more than DMSO and INH treatments at 6-HPI, suggesting that the CF extract could significantly clear the intracellular mycobacteria (Figure 4).

With the observation that CF extract can indeed control intracellular mycobacteria during cellular infection, we next performed a genotypic and phenotypic characterization of *Streptomyces* sp. SbAr007 to determine the bacterial class.

### 3.5. Characterization of Streptomyces sp. SbAr007

#### 3.5.1. Phylogenetic Analysis Revealed *Streptomyces* sp. SbAr007 Clustered with *Streptomyces samsunensis*, *Streptomyces malaysiensis*, and *Streptomyces solisilvae*

**Figure 5:**
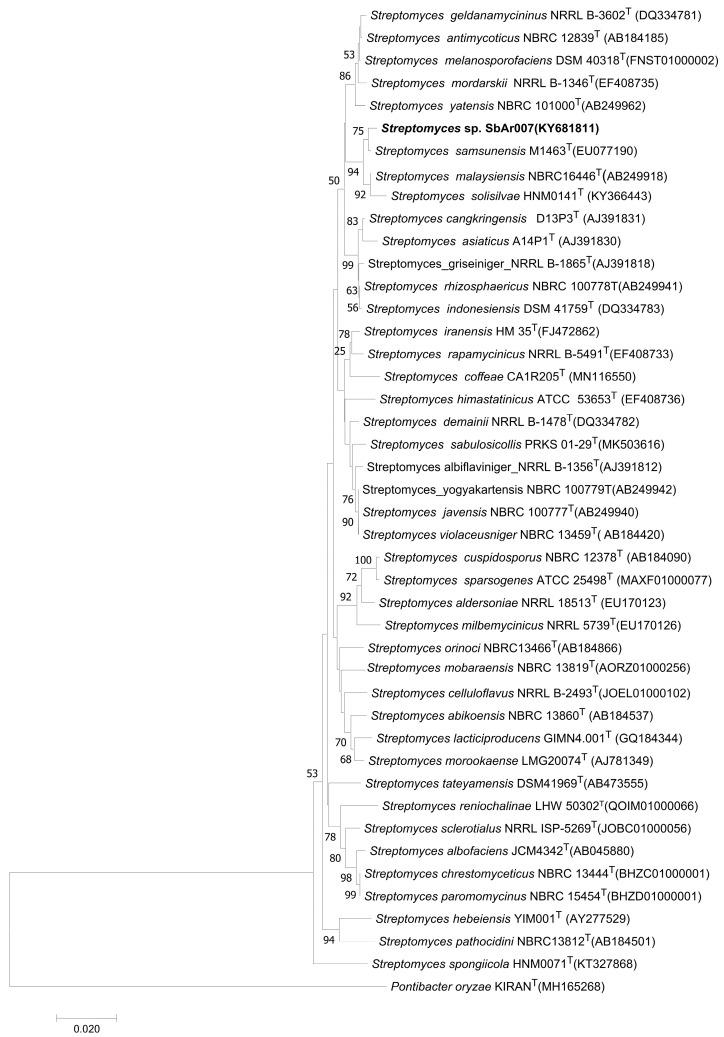
Neighbor-joining phylogenetic tree of *Streptomyces* sp. SbAr007 was generated using the NGPhylogeny.fr open website [39,40] based on the 16S rRNA gene sequence.

The identification and classification of any new bacteria or archaea, either at the genus or the species level, can be obtained from 16S rRNA gene sequencing. As the 16S rRNA gene is highly conserved, it is a commonly used, robust technique with which to identify new strains [36,37]. *Streptomyces* sp. SbAr007 was grown on SCN media and harvested, and genomic DNA was extracted. Extracted genomic DNA was loaded on 1% agarose gel for quality check. The 16S rRNA gene was amplified using PCR. A single amplicon of 1.4Kb was observed on the agarose gel (Supplementary Materials, Figure S2), which was sequenced. The 16S rRNA gene sequence (1417 bp) of *Streptomyces* sp. SbAr007 was submitted to NCBI GenBank (GenBank ID: KY681811.1) and to the EzTaxon website [38] to obtain a list of FASTA sequences from 50 species based on pairwise sequence similarity. A phylogenetic tree was constructed by submitting the FASTA sequences to the NGPhylogeny.fr open website [39,40] with closely related species (Figure 5). *Streptomyces* sp. SbAr007 clustered and formed a well-delineated subclade with *S. samsunensis*, *S. malaysiensis*, and *S. solisilvae*, with a pairwise similarity of 99.50%, 99.00%, and 99.43%, respectively (Figure 5). A comparable threshold cutoff of 98.65% in the sequence similarity is widely used in the prokaryotic classifications [36,37]. Hence, the observed sequence similarities of the 16S rRNA gene confirm that the *Streptomyces* sp. SbAr007 belongs to the genus Streptomyces, with closeness to *S. samsunensis*, *S. malaysiensis*, and *S. solisilvae*. The isolate has been deposited at the Microbial Culture Collection (MCC) at the National Centre for Microbial Resources (NCMR), Pune, India.

#### 3.5.2. Strain Comparison Between the Closely Related Subclades of *Streptomyces* sp. SbAr007

*Streptomyces* sp. SbAr007 was phenotypically and biochemically characterized for features such as carbon and nitrogen utilization, pH and NaCl tolerance, aerial mycelia, melanin formation, tyrosinase production, etc., which are detailed in the supplementary text and Table S2. Some important characteristic features of the *Streptomyces* sp. SbAr007 strain were then compared to *S. samsunensis*, *S. malaysiensis*, and *S. solisilvae*. Table 1 compares some of the phenotypic features of *Streptomyces* sp. SbAr007 using the available literature on *S. samsunensis*, *S. malaysiensis*, and *S. solisilvae*. The table clearly shows that *Streptomyces* sp. SbAr007 has distinguishing features and can be considered a new related isolate belonging to the genus Streptomyces.

### 3.6. Identification of Potential Bioactive Molecules in the Culture Filtrate Extracts of Streptomyces sp. SbAr007

With the promising anti-*M.tb* activity of the CF extract, we proceeded to identify the potential bioactive molecules. To this end, we cultured *Streptomyces* sp. SbAr007 in different batches and pooled all the CF extracts as a single sample and column-fractionated it, as mentioned in the Supplementary Materials (Protocols S1.6: column fractionations of the culture filtrate extract). We attempted to fractionate the culture filtrate extracts via column chromatography. We arrived at a partially pure fraction that showed enhanced anti-*M.tb* activity with MIC_50_ of 3.24 μg/mL against H37Rv, almost 1.6 folds lower than that of CF extract (Supplementary Materials, Figure S3e,f). Though we could arrive at a partially pure fraction with a purity of around 90%, we repeatedly found that we could obtain it in extremely low concentrations for further studies. As the chemical structure elucidation with a very low concentration fraction was challenging with ineffective LC-MS/MS spectra, which can be misleading in obtaining chemically active metabolites, we decided to perform LC-MS of the CF extract and enlist all the molecules present. This was performed in three biological replicates, and a list of metabolites was generated. This has been submitted as a supplementary data file (see the separate Excel sheet entitled “List of metabolites identified from LC-MS data of SbAr007 active culture filtrate extract”).

#### Analysis of Metabolites Obtained in the Active Ethyl Acetate Extracts of *Streptomyces* sp. SbAr007 Culture Filtrates

**Figure 6:**
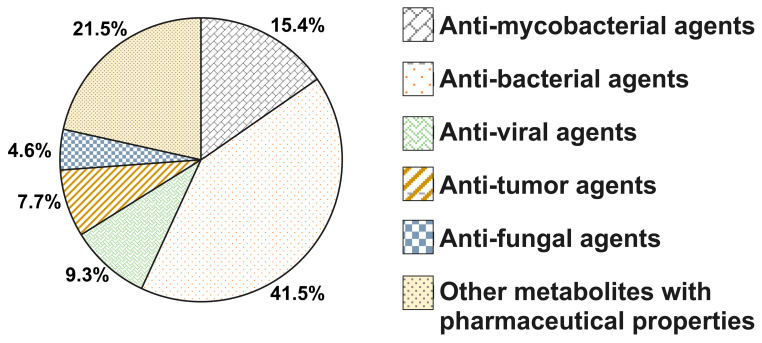
Categorized metabolites of *Streptomyces* sp. SbAr007 culture filtrate extract based on pharmaceutical properties. Other metabolites with pharmaceutical properties include anti-metastatic, anti-proliferative, and immunomodulatory activity, anti-microsporidial activity, treatment of Type 2 diabetes, and cardiovascular diseases, etc.

LC-MS data were subjected to analysis through the MZmine database, as described in material and methods section. Metabolites were identified and listed using *m*/*z* values from HMDB/KEGG databases. Upon obtaining the list, we focused on known chemical entities. It was intriguing to notice a spectrum of known antibiotics and other metabolites with pharmaceutical properties in the extracts. We categorized the known chemical entities into anti-mycobacterial, anti-bacterial, anti-fungal, anti-HIV, anti-cancer, and other metabolites with pharmaceutical properties (Figure 6). Table S3 (Supplementary Materials) enlists these metabolites. Some of the identified metabolites belonging to anti-mycobacterial compounds are norfloxacin [44,45], puromycin [44], levofloxacin [46], sparfloxacin [47], tetracycline [48], gentamicin [49,50], kanamycin A [51], microcystin-LR [52], liposidomycin B [53], and moxifloxacin [54].

Amongst these, liposidomycin B and the liposidomycin class of antibiotics are known to perturb bacterial cell walls. The intramembrane synthesis of the bacterial peptidoglycan layer starts by forming undecaprenyl-P-P-MurNAc-pentapeptide by transferring the phospho-MurNAc-pentapeptide to undecaprenyl phosphate (C55-P) from UDP-MurNAc-pentapeptide. This reaction is catalyzed by phospho-MurNAc-pentapeptide translocase (MraY or translocase I) [53,55]. Liposidomycin B belongs to a class of uridyl lipo-nucleoside antibiotics and is a slow-binding, non-competitive inhibitor to translocase I activity, which was confirmed using a continuous enzymatic assay with a fluorescent analogue, UDPMurNAc-L-g-D-Glu-m-DAP(Nε-dansyl)-D-Ala-D-Ala. MurX, a mycobacterial translocase I, is an ortholog of MraY. Liposidomycin A, class types III and IV, have been shown to inhibit the translocase I activity in *Mycobacterium phlei* [53,55].

In addition, several other metabolites identified in this study are directly or indirectly associated with lowering lipid deposition. Metabolic disorders like dyslipidemia and cardiovascular diseases are linked to lipid accumulation. One study from our group showed that cholesterol-treated macrophages resulted in a foamy phenotype, which resembles the dyslipidemia-like condition, thereby making the macrophage more susceptible to TB infection or persistence [22]. Metabolites such as phloridzin, raffinose, ginsenoside Rg3, and kaempferide were shown to reduce the hepatic lipid and triglyceride accumulation by attenuating the SREBP-1c of the mTORC1 pathway [56,57,58,59,60]. Similarly, tangeretin-treated HepG2 cell lines were shown to decrease the mRNA and protein expression levels of angiopoietin-like protein 3 (ANGPTL3), thereby abolishing the circulating triglycerides in the bloodstream [61]. A few studies involving human subjects administrated with raloxifene, fluvastatin, quercetin, capsaicin, pantethine, taurine, and L-carnitine showed considerable lipid-lowering effects compared to the placebo groups [62,63,64,65,66,67,68]. Many other metabolites, such as gypenoside LXXV, phylloquinone, retinoate, hesperetin, N-acetylcysteine (NAC), estrone, propionate, and monomethylarsonous acid (MMA(III)), etc., are reported to have lipid-reducing properties [69,70,71,72,73,74,75,76,77]. As reported in many other studies, mycobacteria utilizes a cholesterol-rich environment for successful infections [22,78]. This quorum of identified secondary metabolites may further assist the active anti-mycobacterial metabolite to function properly.

While it definitely is a cumulative effect of all known antibiotics on *M.tb*, the presence of other metabolites in the CF extract, especially those that can affect lipid metabolism, may further help in the elimination of *M.tb* during infection. As such kinds of combinations are not used in synthetic drug formulations, extensive investigations via metabolic profiling to identify the best combinations can be performed. In addition, the extract itself can also be developed as a formulation against drug-resistant strains.

## 4. Conclusions

We identified and characterized a novel strain belonging to the genus Streptomyces, closely related to *S. samsunensis*, *S. malaysiensis*, and *S. solisilvae*, the culture filtrate extracts of which showed broad-spectrum anti-mycobacterial activity against pathogenic laboratory *M.tb* strains, a clinical MDR *M.tb* strain, NTMs, and saprophytic environmental mycobacteria. These extracts could effectively reduce intracellular mycobacteria during cellular infection and had negligible toxicity against human primary cells, indicating their safety to the host. With LC-MS showing that it is composed of several chemical entities, both those with antibiotic properties and those effecting lipid biogenesis, this extract has the potential to be developed as a formulation against drug-sensitive and drug-resistant mycobacterial strains. Further, it will be interesting to investigate the stoichiometric ratio of these metabolites, which effectively controlled the growth of the MDR strain.

## Figures and Tables

**Table 1:** Comparison of phenotypic features of the *Streptomyces* sp. SbAr007 with closely related Streptomyces species, *S. samsunensis*, *S. malaysiensis*, and *S. solisilvae.* NA = no data available.

	*Streptomyces* sp. SbAr007	*S. samsunensis* [41]	*S. malaysiensis* [42]	*S. solisilvae* [43]
Sole carbon utilization tests (% *w*/*v*)				
Cellobiose	+	−	+	−
Xylose	+	−	+	+
Sucrose	−	−	−	+
Fructose	+	+	+	NA
D-galactose	+	+	+	+
Mannitol	−	+	+	−
Maltose	+	+	+	−
Dextrose/Glucose	+	+	+	+
L-Rhamnose	+	+	+	NA
Sole nitrogen utilization tests (% *w*/*v*)				
L-arginine	−	+	NA	+
L-asparagine	+	NA	NA	+
Histidine	+	+	+	−
L-Cysteine	−	−	NA	−
Growth at pH 4.0	+	−	−	−
Growth at pH 5 to 10	+	+	NA	+
Growth in NaCl (3%, *w*/*v*)	+	NA	+	+
Growth in NaCl (5%, *w*/*v*)	+	NA	−	+
Growth at 25–30 °C	+	+	+	+
Growth at 37 °C	−	+	+	+
Antibiotic resistance (µg)				
Ampicillin (10)	−	NA	+	NA
Rifampicin (32 and 64)	+/−	NA	NA	+
Gentamicin sulphate (10)	−	+	−	−
Chloramphenicol (30)	−	NA	−	+
Kanamycin (30)	−	NA	−	−

## Data Availability

All relevant data can be found in the manuscript and the Supplementary Materials files.

## Supplementary Materials

The following Supplementary Materials can be downloaded at: https://www.mdpi.com/article/10.3390/tropicalmed10050117/s1: S1: Protocols, drug susceptibility test (DST), preparation of media for actinomycetes strains, morphological and biochemical characterization, phenotypic characterization, biochemical characterization, column fractionations of the *Streptomyces* sp. SbAr007 culture filtrate extract [20,79,80,81,82,83]. Figure S1: Microdilutions assays with different mycobacterial strains; Figure S2: Characterization of *Streptomyces* sp. SbAr007; Figure S3: Column fractionation of *Streptomyces* sp. SbAr007 culture filtrate extracts; Table S1: Drug susceptibility test (DST) [20]; Table S2: Comparative cultural characteristics of Streptomyces sp. SbAr007 strain with *S. samsunensis*, *S. malaysiensis*, and *S. solisilvae* [41,42,43]; Table S3: List of categorized metabolites obtained from LC-MS analysis; References; A list of identified metabolites in triplicates was provided in a separate Excel sheet entitled “List of metabolites identified from LC-MS data of SbAr007 active culture filtrate extract”.
