# Supplementary material for: Secondary Metabolites from a New Antibiotic-Producing Endophytic Streptomyces Isolate Inhibited Pathogenic and Multidrug-Resistant Mycobacterium tuberculosis Strains
Source: Trop Med Infect Dis. 2025 Apr 23;10(5):117. doi: 10.3390/tropicalmed10050117 (PMC12115879; doi:10.3390/tropicalmed10050117)
Supplement: Supplementary file 1 [file tropicalmed-10-00117-s001.zip › tropicalmed-3428274-supplementary.pdf]

Supplementary Materials

Secondary Metabolites from a New Antibiotic-Producing Endophytic Streptomyces Isolate Inhibited Pathogenic and Multidrug-Resistant *Mycobacterium tuberculosis* Strains

Govinda Raju Vadankula [1,†], Arshad Rizvi [1,†,‡], Haider Ali [1,§,‖], Rakhi Khunjamayum [2,‖], V. V. Ramprasad Eedara [3,‖], Vijay Nema [4,¶], Debananda Singh Ningthoujam [2], Katragadda Suresh Babu [5], Prakasham Reddy Shetty [6], Shekhar C. Mande [7,8] and Sharmistha Banerjee [1,*]

[1] Laboratory of Molecular Pathogenesis, Department of Biochemistry, School of Life Sciences, University of Hyderabad (UoH), Hyderabad 500046, India; govindraj.vadankula@gmail.com (G.R.V.); arshad.rizvi@emory.edu (A.R.); ali.haider@doctoral.uj.edu.pl (H.A.)
[2] Microbial Biotechnology Research Laboratory (MBRL), Department of Biochemistry, Manipur University, Canchipur 795003, India; rakhikhunjamayum@gmail.com (R.K.); debananda.ningthoujam@gmail.com (D.S.N.)
[3] MNR Medical College and Hospital, MNR Nagar, Fasalwadi, Narsapur Road, Sanga Reddy, Hyderabad 502294, India; ramprasadevv@gmail.com
[4] Molecular Biology Division, National Institute of Translational Virology and AIDS Research (Formerly National AIDS Research Institute), Pune 411026, India; dr.vijaynema@gmail.com
[5] Centre for Natural Products and Traditional Knowledge, CSIR-Indian Institute of Chemical Technology, Hyderabad 500007, India; suresh@iict.res.in
[6] Medicinal Chemistry and Biotechnology Lab- Organic Synthesis and Process Chemistry Division, CSIR-Indian Institute of Chemical Technology, Hyderabad 500007, India; prakasham@iict.res.in
[7] National Centre for Cell Science, Pune 411007, India; shekhar.mande@gmail.com
[8] Bioinformatics Centre, Savitribai Phule Pune University, Pune 411007, India
[*] Correspondence: sbsl@uohyd.ac.in
[†] These authors contributed equally to this work.
[‡] Current address: Department of Microbiology and Immunology, Emory University School of Medicine, Emory Antibiotic Resistance Centre, Atlanta, GA 30322, USA.
[§] Current address: Molecular Virology Group, Malopolska Centre of Biotechnology, Jagiellonian University, Gronostajowa 7A Str., 30-387 Kraków, Poland.
[‖] These authors also contributed equally to this work.
[¶] Current address: ICMR-National Institute of Research in Tribal Health, ICMR-NIRTH Campus, Nagpur Rd, Dhanvantri Nagar, Garha, Jabalpur 482003, India.

Table of contents

Description	*Page number*
S1: Protocols	***S2-S4***
S1.1.) Drug susceptibility test (DST)	*S2*
S1.2.) Preparation of media for actinomycetes strains	*S2*
S1.3.) Morphological and Biochemical characterization	*S2-S3*
S1.4.) Phenotypic characterization	*S3*
S1.5.) Biochemical characterization	*S3-S4*
S1.6.) Column fractionations of the Streptomyces sp. SbAr007 culture filtrate extract	*S4*
Figure S1: Microdilutions assays with different mycobacterial strains	S5
Figure S2: Characterization of Streptomyces sp. SbAr007	S6

Figure S3: Column fractionation of Streptomyces sp. SbAr007 culture filtrate extracts	S7
Table S1: Drug susceptibility test (DST)	S8
Table S2: Comparative cultural characteristics of Streptomyces sp. SbAr007 strain with S. samsunensis, S. malaysiensis, and S. solisilvae	S8-S9
Table S3: List of categorized metabolites obtained from LC-MS analysis	S9
References	S10

S1. Protocols

S1.1.) Drug susceptibility test

DST assays were performed in a BACTEC™ MGIT™ 960 machine (BD, USA), as described [1]. The susceptibilities of the MDR-*M.tb* (CH56) and *M.tb* H37Rv strains were tested against the antibiotics streptomycin (S), isoniazid (I), rifampicin (R), and ethambutol (E) using SIRE Kit (BD, NJ, USA) and growth controls (GC) tubes, without any drug for the respective strains and for every set of experiments as per the Clinical and Laboratory Standards Institute (CLSI) standards. Bacterial inoculum was prepared from fresh LJ slants as described. Inoculated tubes were incubated for 3 to 4 weeks, and the growth status was monitored and recorded in real time using a BACTEC™ MGIT™ 960 machine.

S1.2.) Preparation of media for actinomycetes strains

The media for culturing actinomycetes strains were prepared, as described [2,3].

Starch Casein Nitrogen (SCN) media: Starch casein nitrate agar media were prepared by mixing 1mg of starch, 0.03gm of casein, 0.2gm each of NaCl, KNO_3, and K_2HPO_4, 0.005gm of $MgCl_2$, 0.002gm of $CaCO_3$, 0.001gm of $FeSO_4$, and the final volume was adjusted to 100ml with MilliQ water. After the pH was set to 7.25, agar (1.8gm) was added for preparing SCN–agar media, whereas no agar was added for preparing SCN–broth media and sterilized for 20 minutes at 120°C.

ISP media: The following media ingredients were added to 100ml of MilliQ water separately in their dedicated flasks: ISP1 (pancreatic digest of casein and yeast extract (0.5gm and 0.3gm, respectively)); ISP2 (yeast extract malt extract agar), 4.1gm; ISP3 (oatmeal agar medium), 3.8gm; ISP4 (inorganic salts agar medium), 3.7gm; ISP5 (glycerol–asparagine agar medium), 2.3gm; ISP6 (peptone yeast extract iron agar medium), 3.758gm; ISP7 (tyrosine agar medium), 2.3gm; and glycerol, 1.5ml. The pH was set to 7.25 and sterilized for 20 minutes at 120°C.

Basal Mineral Salts Agar: A total of 2.64gm of $(NH_4)_2SO_4$, 2.38gm of anhydrous KH_2PO_4, 1gm of $MgSO_4.7H_2O$, 1ml of Pridham and Gottlieb trace salts (which is prepared by dissolving $CuSO_4.5H_2O$ (0.64gm), $FeSO_4.7H_2O$ (0.11gm), $MnCl_2.4H_2O$ (0.79gm), and $ZnSO_4.7H_2O$ (0.15gm) in 100ml of MilliQ water) was added in 100ml of MilliQ water and sterilized for 20 minutes at 120°C.

S1.3.) Morphological and Biochemical characterization

The International Streptomyces Project procedures [2] were used for morphological and cultural studies. Different ISP plates were prepared, as described above, and empty plates were incubated at 37°C for 24 hours to check for contamination. After 24 hours, *Streptomyces* sp. SbAr007 was streaked and incubated for 14 to 20 days. Next, the color of aerial mycelium, substrate mycelium, and soluble pigment was observed and recorded by the naked eye.

Carbon utilization test: Carbon utilization was checked by growing the *Streptomyces* sp. SbAr007 in Pridham and Gottlieb trace salts medium in the presence of 0.01% of each carbon source. A total of 16 different carbon sources were used in our study, namely, galactose, D-ribose, sucrose, glucose, D(+)

xylose, maltose, lactose, L(+) rhamnose monohydrate, D(+) mannose, D(+) cellobiose, D(+) fructose, D(+) arabinose, mannitol, sorbitol, glycerol, and D(-) ribose. Changes in the color of phenol red (pH indicator) were recorded, and the growth of the test isolate was assessed by comparing the color change with the following controls: basal media alone, basal media with phenol red, and basal media inoculated with test strain.

Nitrogen utilization test: Nitrogen utilization was checked by growing the *Streptomyces* sp. SbAr007 in a Pridham and Gottlieb trace salts medium in the presence of 1% of each nitrogen source and phenol red (pH indicator). Potassium nitrate, L- asparagine, arginine, and histidine were used as nitrogen sources. Changes in the color were recorded, and the growth of the test isolate was assessed until the 14th day by comparing the color change with the following controls: basal media alone, basal media with phenol red, and basal media inoculated with test strain.

Melanin Formation: Formation of melanin was observed on ISP-1, ISP-6, and ISP-7 media. Production of deep-brown, greenish-brown, greenish-black, or black by mature streptomyces strain is considered melanin positive. The absence of brown to black color or the diffusible pigment was considered as negative for melanin formation.

Tyrosine reaction: Tyrosine reaction was observed on ISP-7 (tyrosine agar medium). The strain was streaked on ISP-7 and incubated at 30°C for 15 to 20 days. Production of greenish-brown, brown, bluish-black, and black diffusible pigment was considered tyrosinase positive.

Sodium Chloride Tolerance test: Sodium chloride tolerance was determined by growing the *Streptomyces* sp. SbAr007 on an SCNA medium supplemented with an increasing concentration of sodium chloride from 0.5% to 10%. The SCNA plates were streaked with the isolated strain suspension and incubated at 30°C for 7 to 14 days, and the growth was monitored and recorded.

pH-sensitive test: The ability of *Streptomyces* sp. SbAr007 to grow at different pH levels was observed by using an SCNA medium with different pH ranges from 4 to 13. The SCNA plates were streaked with *Streptomyces* sp. SbAr007 and incubated at 30°C for 7 to 14 days, and the growth was monitored and recorded.

Antibiotic sensitive test: The sensitivity or resistance of *Streptomyces* sp. SbAr007 to different drug pressures was investigated using the disc diffusion method as per Clinical and Laboratory Standards Institute protocols [4]. SCNA plates were streaked with *Streptomyces* sp. SbAr007, and sterile antibiotic discs were placed on the streaked plates and incubated at 30°C for 7 to 14 days. A battery of antibiotics was used in this study, including ciprofloxacin (0.01-240µg), gentamycin (10µg), streptomycin (10µg), tetracycline (30µg), ampicillin (10µg), kanamycin (30µg), co-trimoxazole (25µg), amikacin (30µg), chloramphenicol (30µg), trimethoprim (2.5µg), sulphamethxazole (50µg), rifampicin (0.001-240µg), cefuroxime (30µg), cephotaxime (30µg), cefoperazone (75µg), ampicillin/sulbactam (10/10µg), piperacillin/tazobactum (100/10µg), ticarcillin/clavulinic acid (75/10µg), carbenicillin (100µg), gatifloxacin (5µg), nitrofurantoin (300µg), levofloxacin (5µg), azotreonam (30µg), ofloxacin (5µg), and morfloxacin (10µg). The growth was monitored and recorded.

S1.4.) Phenotypic characterization

Streptomyces sp. SbAr007 was grown on different ISP media (ISP 1-7) that differ in their constituents, as described above. *Streptomyces* sp. SbAr007 grew well on ISP media such as tryptone yeast extract agar media (ISP-1), oatmeal agar media (ISP-3), inorganic salts agar media (ISP-4), glycerol–asparagine agar media (ISP-5), peptone yeast extract iron agar media (ISP-6), and tyrosine agar medium (ISP-7), whereas no growth was observed in yeast extract maltose agar media (ISP-2) (Table S2). It forms different aerial mycelia, mostly white in ISP-1, 4, 5, and 7, brown in ISP6, and smoky-black in ISP3. However, the substrate mycelia (reverses color) were observed as yellow in ISP-3 and 6, light yellow in ISP7, and yellow-brown in ISP-1, 4, and 5. When *Streptomyces* sp. SbAr007 is incubated on different ISP media, like ISP-1, ISP-6, and ISP-7, for 20 days, the matured strain produced smoky-black, brown, and

greenish-black pigmentation, respectively and was recorded as melanin-positive. Thus, *Streptomyces* sp. SbAr007 is positive for melanin formation and tyrosinase enzyme production [2,3,5,6].

S1.5.) Biochemical characterization

Carbon utilization test: A carbon utilization test was performed to check the growth status of the *Streptomyces* sp. SbAr007 in 16 different carbon sources, namely, galactose, D-ribose, sucrose, D-glucose, D(+) xylose, maltose, lactose, L(+) rhamnose monohydrate, D(+) mannose, D(+) cellobiose, D(+) fructose, D(+) arabinose, mannitol, sorbitol, glycerol, and D(-) ribose, using phenol red as the indicator. Except for sucrose and sorbitol, *Streptomyces* sp. SbAr007 grew in the rest of the carbon sources, suggesting that it is not utilizing sucrose and sorbitol as a carbon source (Table S2).

Nitrogen utilization test: Similarly, *Streptomyces* sp. SbAr007 was tested for nitrogen utilization with five different nitrogen sources, such as L-arginine, potassium nitrate, L-asparagine, and histidine, using phenol red as the indicator. It was grown in L-asparagine- and histidine-containing media; however, it did not show any growth in L-arginine- and potassium-nitrate-containing media, indicating that it was not able to utilize L-arginine and potassium nitrate as a nitrogen source (Table S2).

NaCl tolerance test: Furthermore, *Streptomyces* sp. SbAr007 is grown on different concentrations of sodium chloride (NaCl) to determine its tolerance towards the NaCl. It grew in NaCl-containing media within a range of 0.5% to 5%. However, higher concentrations of NaCl above 8% did not allow SbAr007 to grow, which hints that its tolerance to 5% NaCl and concentrations above this are lethal to the bacteria (Table S2).

pH tolerance: Next, *Streptomyces* sp. SbAr007 was grown on pH ranging from 4 to 13. Growth of *Streptomyces* sp. SbAr007 was observed in pH 4, 5, 9, 10, and 11, whereas pH 7 and pH 8 showed maximum growth. However, no growth was observed in pH 12 and pH 13. From this, it can be inferred that the optimum growth for *Streptomyces* sp. SbAr007 is from pH 4 to 11, with a maximum growth at pH 7 and pH 8 (Table S2).

S1.6.) Column fractionations of the Streptomyces sp. SbAr007 culture filtrate extract

The slurry to pack the column was prepared using silica with a mesh size of 60-100. Silica was soaked in dichloromethane (DCM) and packed in the column. The column was saturated with DCM, and the *Streptomyces* sp. SbAr007 culture filtrate extract was loaded onto the column and allowed to fractionate, using 0.5% of methanol in DCM as a solvent. All the elutes were collected immediately. The methanol percentage was gradually increased to 5% to elute the pure fraction. Each fraction was checked on TLC, and bands with similar patterns were pooled together. We collected different fractions and concentrated them using rotavapor. All the fractions were pooled into 25 fractions according to their band patterns and concentrated using rotavapor. TLC profiles of all the fractions were recorded and a representative TLC is depicted below (Figures S3a), where rifampicin (R) and streptomycin (S) were used as standards. The anti-mycobacterial activity was checked for a few fractions against *M.tb* H37Rv using a microdilution (MTT) assay. Except for fraction Y_{40}, no other tested fractions have shown activity against pathogenic mycobacteria. MIC_{50} for fraction Y_{40} was calculated and found to be 3.24µg/ml (Figures S3e and S3f). Qualitative analysis was performed using silica gel thin-layer chromatography. Fractionation of culture filtrate extract was carried out by HPLC on a C18 column with acetonitrile–water. The HPLC was run with a flow rate of 1ml/ml in a binary gradient for 60 minutes. The mobile phase of Milli-Q water (A) and acetonitrile (B) was used, and the absorbance was recorded at 310nm. We observed a peak at a retention of 22 minutes with a maximum intensity. However, a few peaks of impurities were also isolated along with the compound of our interest. We could not perform the effective LCMS for the same sample Y_{40} to identify the potential bioactive molecules as the fraction was obtained in very minute concentrations. Hence, we decided to enlist all the molecules in the culture filtrate extract using LC-MS analysis.

Figure S1

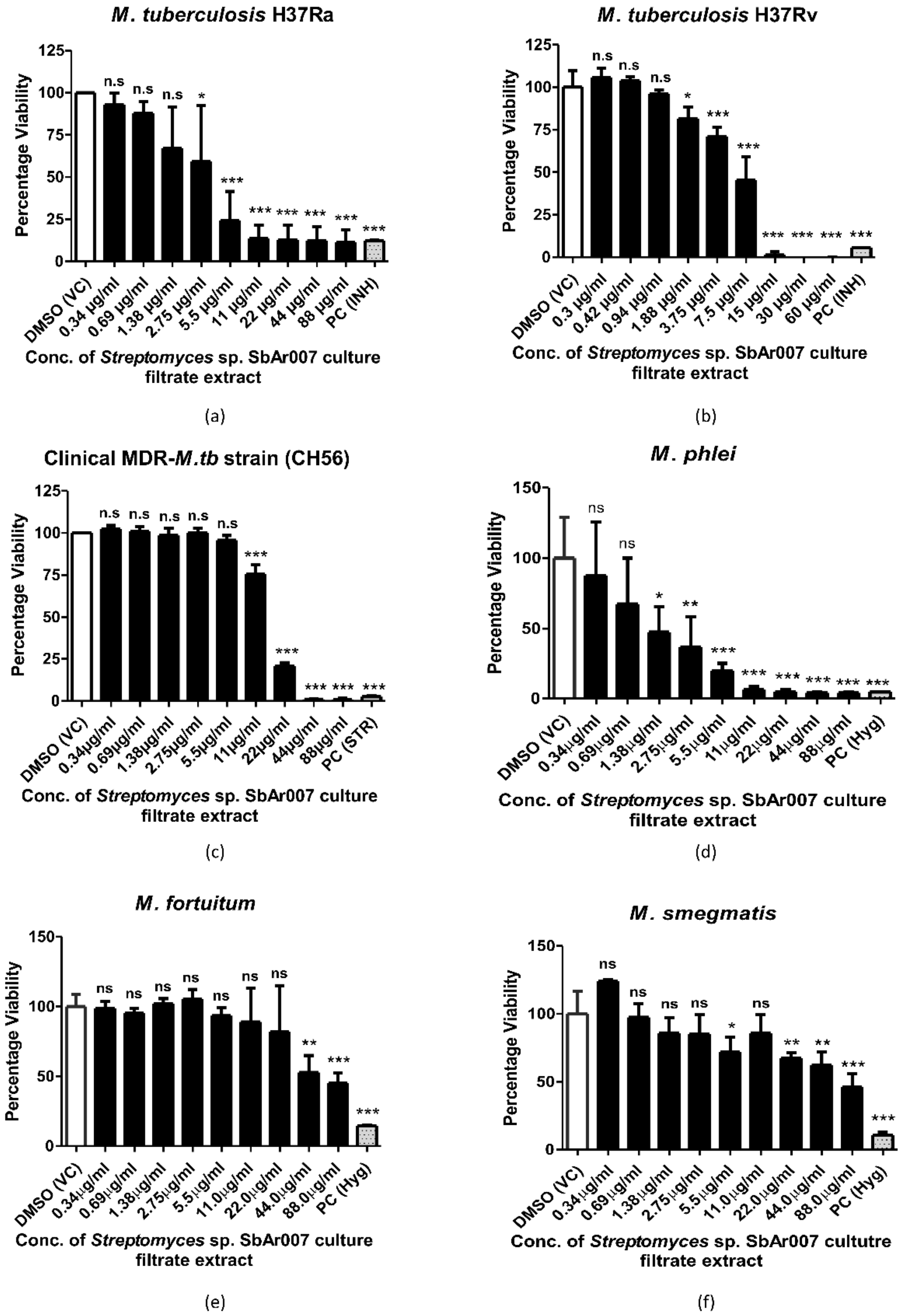

Figure S1. Microdilution assays of various mycobacterial strains. These assays are used to determine MIC$_{50}$ upon treatment with *Streptomyces* sp. SbAr007 culture filtrate extract against (a) *M. tuberculosis* H37Ra, (b) *M. tuberculosis* H37Rv, (c) a representative clinical MDR-*M. tuberculosis* strain (CH56) (resistant to established anti-TB drugs, isoniazid and rifampicin), (d) *M. phlei*, (e) *M. fortuitum*, and (d) *M. smegmatis*. Positive control (PC) used in this study: isoniazid (INH), 0.2µg/ml; streptomycin (STR), 1µg/ml; hygromycin (Hyg), 50µg/ml. One-way ANOVA (Dunnett's multiple comparison test) was performed, and the error bars represent the standard deviation. "***" denotes a *p*-value of < 0.001, "**" denotes a *p*-value of < 0.01, "*" denotes a *p*-value of < 0.05, and "ns" denotes a *p*-value of > 0.05.

Figure S2

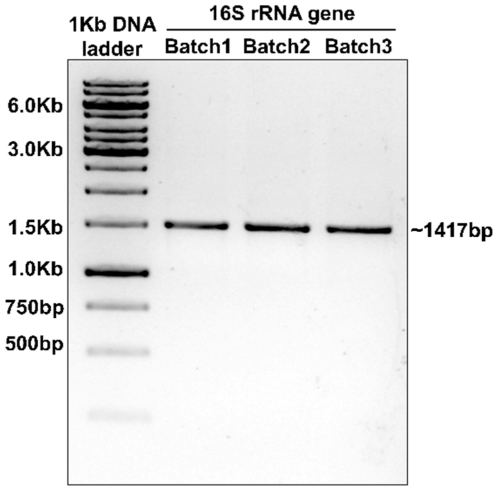

Figure S2: Characterization of *Streptomyces* sp. SbAr007: 16S rRNA gene amplification from isolated *Streptomyces* sp. SbAr007 genomic DNA.

Figure S3

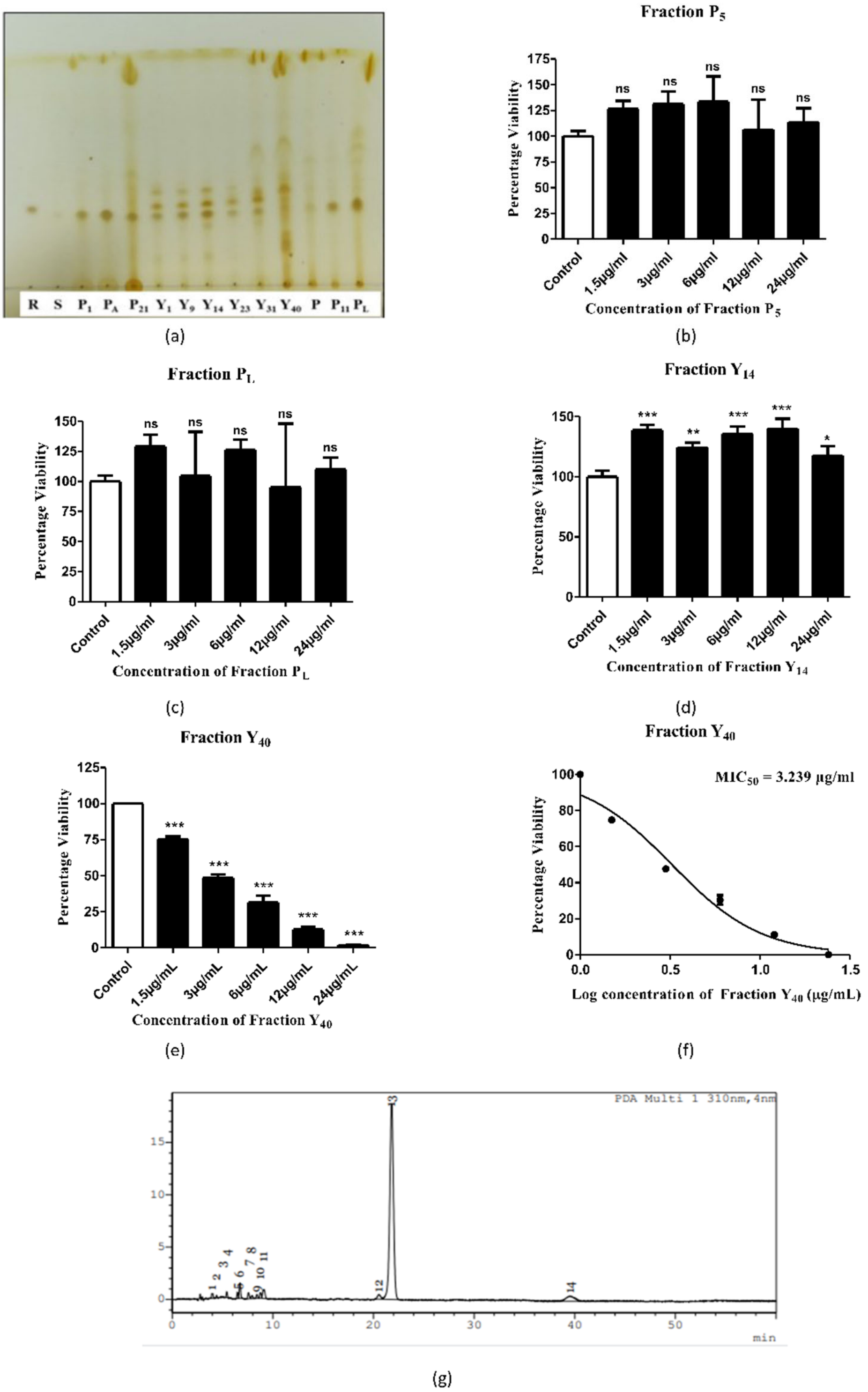

Figure S3: Column fractionation of *Streptomyces* sp. SbAr007 culture filtrate extract: (a) TLC profiling of a few representative fractions purified using column fractionation. R: rifampicin; S: streptomycin. P_1, P_A, P_{21}, Y_1, Y_9, Y_{14}, Y_{23}, Y_{31}, Y_{40}, P, P_{11}, and P_L represent different fractions. Microdilution (MTT) assays of (b) fraction P_5, (c) fraction P_L, (d) fraction Y_{14}, and (e) fraction Y_{40} obtained from column fractionation. (f) Dose–response curve of fraction Y_{40}. (g) HPLC of column fractionated fractions. One-way ANOVA (Dunnett's multiple comparison test) was performed, and the error bars represent the standard deviation. "***" denotes a p-value of < 0.001. "**" denotes a p-value of < 0.01, "*" denotes a p-value of < 0.05, and "ns" denotes a p-value > 0.05.

Table S1: Drug susceptibility test on H37Rv and MDR-*M.tb* (CH56) strains with established anti-TB drugs showing the growth status of the mycobacteria using BACTEC [1]. C: control; S: susceptible; R: resistant; DS: drug-susceptible; DR: drug-resistant; MDR: multidrug-Resistant.

**Drug tesed**	**Concentraion used (µg/ml)**	***M.tb* Strain**	
		**H37Rv**	**CH56**
Growth control	---	C	C
Streptomycin	1	S	S
Isoniazid	0.1	S	R
Rifampicin	1	S	R
Ethambutol	5	S	S
Inference		DS	MDR

Table S2: Comparative cultural characteristics of *Streptomyces* sp. SbAr007 strain with *S. samsunensis*, *S. malaysiensis*, and *S. solisilvae*. +++, ample growth; ++, modest growth; +, less growth.

**Properties**	***Streptomyces* sp. SbAr007**	***S. samsunensis*** [7]	***S. malaysiensis*** [8]	***S. solisilvae*** [9]
ISP1				
Growth	+++	+++	++	+
Aerial mycelia	White	White-grey	Grey	Grey
Substrate mycelia (reverse color)	Yellow-Brown	Greenish White	Light Yellow	Yellow-brown
ISP2				
Growth	-	+++	+++	+++
Aerial mycelia		Grey	Dark-grey	Grey
Substrate mycelia (reverse color)		Brown	Brown-grey	Dark-grey
ISP3				
Growth	+++	+++	+++	+++
Aerial mycelia	Smoky-Black	Grey	Smoky-Black	White-grey
Substrate mycelia (reverse color)	Yellow	Greyish-Yellow	Yellow-Brown	Yellow-green
ISP4				
Growth	+++	+++	+++	+++
Aerial mycelia	White	Grey	Smoky-Black	Pinkish grey
Substrate mycelia (reverse color)	Yellow-Brown	Greyish-Green	Dark-Grey	Yellow-green
ISP5				

Growth	++	+++	+++	+++
Aerial mycelia	White	Grey	White-grey	Grey
Substrate mycelia (reverse color)	Yellow-Brown	Brown	Pale-yellow-grey	Light yellow
ISP6				
Growth	+	+++	+++	+++
Aerial mycelia	Brown	Grey	Grey	Grey
Substrate mycelia (reverse color)	Yellow	Brown	Brown	Brown-grey
ISP7				
Growth	+++	+++	+++	+++
Aerial mycelia	White	Grey	Grey	Grey
Substrate mycelia (reverse color)	Light-Yellow	Brown	Brown	Yellow-green

Table S3. List of categorized metabolites obtained from LC-MS analysis.

	List of Metabolites
Anti-mycobacterial agents	Norfloxacin, Levofloxacin, Sparfloxacin, Tetracycline, Gentamicin A, Puromycin, Kanamycin A, Microcystin-LR, Liposidomycin B, Moxifloxacin
Anti-bacterial agents	Cefaloglycin, Cephalosporin C, Cefazolin, Gentamicin A2, Oxytetracycline, Lincomycin hydrochloride, Gentamicin C2, Tetracenomycin C, Netilmicin, Gentamicin C1, Gentamicin X2, Mupirocin, Ceforanide, Moxalactam, Apramycin, Jadomycin B, Rebeccamycin, Naringin, Lividomycin B, Paromomycin, Rifamycin W, Elloramycin A, Mycinamicin VI, Erythromycin D, Pheophytin a, Coumermycin D, Anemone blue anthocyanin 1 and 2
Anti-viral agents	Deoxymannojirimycin, Zalcitabine, Acyclovir, Indinavir, Nelfinavir mesylate, Eugeniin
Anti-tumor agents	Ansamitocinoside P-3, Ginsenoside Rg3, Vinblastine, Premithramycin A3', Premithramycin B
Anti-fungal agents	Miconazole, Validamycin A, Nystatin
Other metabolites with pharmaceutical properties	Swainsonine, Sapropterin, Linatine, Albendazole, Tolbutamide, Arbutin, Azathioprine, Tetrabenazine, Betanidin, Fluvastatin, Fumagillin, Zeaxanthin, Digoxin, Gypenoside LXXV

References:

1. Vadankula, G.R.; Nilkanth, V. V; Rizvi, A.; Yandrapally, S.; Agarwal, A.; Chirra, H.; Biswas, R.; Arifuddin, M.; Nema, V.; Mallika, A.; et al. Confronting Tuberculosis: A Synthetic Quinoline-Isonicotinic Acid Hydrazide Hybrid Compound as a Potent Lead Molecule Against Mycobacterium Tuberculosis. *ACS Infect Dis* **2024**, *10*, 2288–2302, doi:10.1021/acsinfecdis.4c00277.
2. Shirling, E.B.; Gottlieb, D. Methods for Characterization of Streptomyces Species1. *Int J Syst Evol Microbiol* **1966**, *16*, 313–340, doi:10.1099/00207713-16-3-313.
3. Pridham, T.G.; Gottlieb, D. The Utilization of Carbon Compounds by Some Actinomycetales as an Aid for Species Determination. *J Bacteriol* **1948**, *56*, 107–114, doi:10.1128/JB.56.1.107-114.1948.
4. Woods, G.L.; Brown-Elliott, B.A.; Conville, P.S.; Desmond, E.P.; Hall, G.S.; Lin, G.; Pfyffer, G.E.; Ridderhof, J.C.; Siddiqi, S.H.; Richard J. Wallace, J.; et al. Susceptibility Testing of Mycobacteria, Nocardiae, and Other Aerobic Actinomycetes. *Susceptibility Testing of Mycobacteria, Nocardiae, and Other Aerobic Actinomycetes* **2011**, *Report No.: M24-A2*.
5. Rudrappa, M.; Kumar M, S.; Kumar, R.S.; Almansour, A.I.; Perumal, K.; Nayaka, S. Bioproduction, Purification and Physicochemical Characterization of Melanin from Streptomyces Sp. Strain MR28. *Microbiol Res* **2022**, *263*, 127130, doi:10.1016/J.MICRES.2022.127130.
6. El-Naggar, N.E.A.; El-Ewasy, S.M. Bioproduction, Characterization, Anticancer and Antioxidant Activities of Extracellular Melanin Pigment Produced by Newly Isolated Microbial Cell Factories Streptomyces Glaucescens NEAE-H. *Sci Rep* **2017**, *7*, 1–19, doi:10.1038/srep42129.
7. Sazak, A.; Şahin, N.; Güven, K.; Işik, K.; Goodfellow, M. Streptomyces Samsunensis Sp. Nov., a Member of the Streptomyces Violaceusniger Clade Isolated from the Rhizosphere of Robinia Pseudoacacia. *Int J Syst Evol Microbiol.* **2011**, *61*, 1309–1314, doi:doi.org/10.1099/ijs.0.021329-0.
8. Al-Tai, A.; Bongcheol, K.; Seung Bum, K.; Manfio, G.P.; Goodfellow, M. Streptomyces Malaysiensis Sp. Nov., a New Streptomycete Species with Rugose, Ornamented Spores. *Int J Syst Bacteriol.* **1999**, *49*, 1395–1402, doi:10.1099/00207713-49-4-1395.
9. Zhou, S.; Yang, X.; Huang, D.; Huang, X. Streptomyces Solisilvae Sp. Nov., Isolated from Tropical Forest Soil. *Int J Syst Evol Microbiol.* **2017**, *67*, 3553–3558, doi:10.1099/IJSEM.0.002166.
